# Within-Host Genomic Diversity of *Candida albicans* in Healthy Carriers

**DOI:** 10.1038/s41598-019-38768-4

**Published:** 2019-02-22

**Authors:** Emilie Sitterlé, Corinne Maufrais, Natacha Sertour, Matthieu Palayret, Christophe d’Enfert, Marie-Elisabeth Bougnoux

**Affiliations:** 1 Fungal Biology and Pathogenicity Unit, Department of Mycology, Institut Pasteur, INRA, Paris, France; 2 Université Paris Diderot, Sorbonne Paris Cité, Cellule Pasteur, Paris, France; 3 Unité de Parasitologie-Mycologie, Service de Microbiologie clinique, Hôpital Necker-Enfants-Malades, Assistance Publique des Hôpitaux de Paris (APHP), Paris, France; 4 Center for Bioinformatics, BioStatistics and Integrative Biology (C3BI), USR 3756 IP CNRS, Institut Pasteur, Paris, France; 5 Department of Biology, Ecole Polytechnique, Palaiseau, France

## Abstract

Genomic variations in *Candida albicans*, a major fungal pathogen of humans, have been observed upon exposure of this yeast to different stresses and experimental infections, possibly contributing to subsequent adaptation to these stress conditions. Yet, little is known about the extent of genomic diversity that is associated with commensalism, the predominant lifestyle of *C. albicans* in humans. In this study, we investigated the genetic diversity of *C. albicans* oral isolates recovered from healthy individuals, using multilocus sequencing typing (MLST) and whole genome sequencing. While MLST revealed occasional differences between isolates collected from a single individual, genome sequencing showed that they differed by numerous single nucleotide polymorphisms, mostly resulting from short-range loss-of-heterozygosity events. These differences were shown to have occurred upon human carriage of *C. albicans* rather than subsequent *in vitro* manipulation of the isolates. Thus, *C. albicans* intra-sample diversity appears common in healthy individuals, higher than that observed using MLST. We propose that diversifying lineages coexist in a single human individual, and this diversity can enable rapid adaptation under stress exposure. These results are crucial for the interpretation of longitudinal studies evaluating the evolution of the *C. albicans* genome.

## Introduction

All living organisms must adapt in order to thrive within their natural niches. They must also be able to rapidly adopt strategies to adapt and survive under stressful conditions. Many fungal pathogens have evolved a highly plastic genome, thereby enabling the generation of genomic diversity^1^. Because *C. albicans* is one of the leading fungal pathogens of humans, genome analysis and the mechanisms that allow this yeast to persist in humans have attracted interest. *C. albicans* is the most common fungal commensal of humans and is considered a facultative component of the normal human digestive microbiota^2–4^. This yeast is also a major opportunistic pathogen responsible for both superficial and disseminated infections in immunocompromised patients^5,6^. In these patients, infections frequently originate from an endogenous source, mainly the digestive tract, which represents the major reservoir of this yeast^7,8^. Therefore, the human digestive tract is probably the most relevant niche to investigate genome diversification in this species.

*C. albicans* is predominantly diploid and demonstrates a significant degree of genetic diversity across isolates, notably variations in the distribution of heterozygous polymorphisms along the genome^9–13^. At the population level, molecular typing has revealed that *C. albicans* strains belong to five major and thirteen minor genetic clusters^14,15^. Some of these clusters exhibit geographical enrichment or phenotypic specificities^14–24^. Yet, no correlation between cluster assignment and the ability of strains to cause different forms of infection has been established^14^.

In addition to heterozygosity, the genome of *C. albicans* displays a high level of plasticity^25^. While *C. albicans* is an asexual organism with a predominantly clonal mode of reproduction^26^, it can also employ a parasexual cycle^10,26–29^. This cycle allows *C. albicans* to alternate between diploid and tetraploid states independently of meiosis, and is frequently accompanied by the generation of aneuploidies and mitotic recombination (and consequently loss-of-heterozygosity [LOH] events) between chromosome homologs^26,27,29^. LOH events are also observed during clonal propagation of diploid isolates, and the mechanisms that underlie these events have been well-studied^25,30^. Interestingly, it has been shown that environmental modifications such as oxidative stress, high temperature, ultraviolet light, or exposure to antifungal agents increase the rate of LOH events or induce ploidy variations in the *C. albicans* genome^26,31,32^. Genomic rearrangements have also been detected *in vivo*, for instance during a passage in mice, which is not the natural host of *C. albicans*^33–35^. In humans, a study conducted by Ford *et al*. (2015) analysing the whole genome of 43 *C. albicans* isolates has revealed that these events were also common during the course of an oral infection^36^. Under these different stressful conditions, both aneuploidy and LOH events can arise quickly, enabling *C. albicans* to survive and possibly adapt to changing environments^1^. However, little is known about the occurrence and diversity of such genetic variations within the natural host when *C. albicans* is a commensal. Studies using molecular typing have shown that *C. albicans* strains can persist in healthy individuals for many years^37,38^ and evolve through minor genetic variations^37,39–45^. To broaden our understanding on the genomic diversity within the healthy host, we addressed the question of the genome-wide genetic heterogeneity between several *C. albicans* isolates obtained from a healthy individual. Our study indicated that genetically distinct and yet closely related isolates co-exist in a healthy individual. This intrinsic within-host genomic diversity should be taken into consideration when evaluating the genomic evolution of *C. albicans* in longitudinal studies, as these studies often characterise a single isolate at any time point.

## Results

### Oral *C. albicans* carriage in healthy individuals

We screened 56 undergraduate students to evaluate the prevalence of oral *Candida* carriage in healthy individuals. Ten of the 56 students (17.9%) were carriers of *Candida* spp., 8 harboured only *C. albicans*, and 2 harboured both *C. albicans* and *C. glabrata*. The prevalence of oral *C. albicans* carriage in this healthy population was 17.9%.

Up to 8 *C. albicans* isolates (colonies) from each carrier were analysed using MLST. As shown in Supplementary Table S1, 10 diploid sequence types (DSTs) belonging to 5 genetic clusters were identified among the 49 isolates analysed from the 10 carriers. Among the 7 students for which several isolates were typed, only one (student G) had isolates that displayed different DSTs. Indeed, isolate G1 differed from isolates G2–G8 at one locus (*ZWF1*). Sequence comparisons of the *ZWF1* locus of these isolates showed that the variations between G1 and G2–G8 resulted from a LOH event involving 5 heterozygous positions (Table S1). Overall, our MLST analysis, while suggesting limited within-host genetic diversity, indicated that *C. albicans* isolates collected from a single carrier in the oral niche could show some genetic diversity, consistent with previous studies^37,39–41^.

### Oral samples harbour genetically diverse *C. albicans* isolates

**Figure 1:**
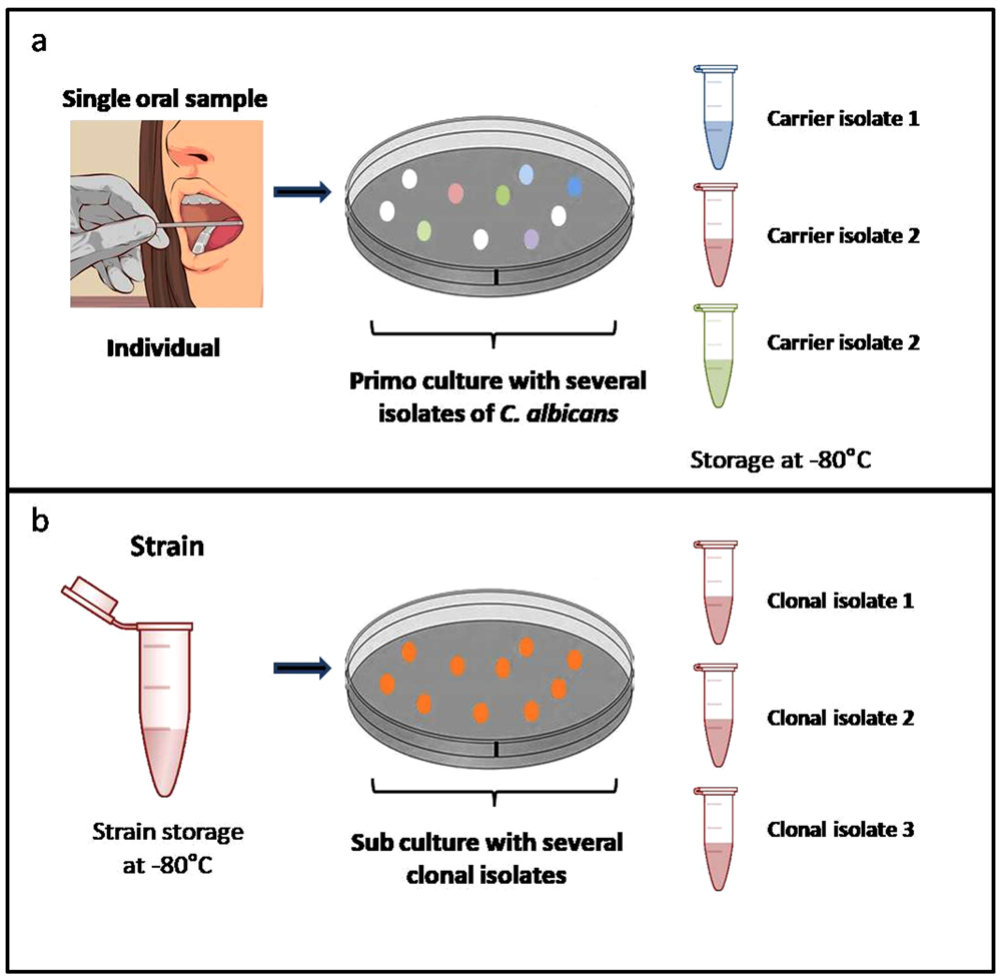
Protocol of the *C. albicans* genomic diversity analysis. (**a**) Genomic diversity between “carrier isolates”. In this context, 3 different “carrier isolates” were selected on the “primo culture” of the single oral swabbing of the individual. The whole genome of the 3 isolated colonies was analysed in order to determine the population genomic diversity within single oral sample. In total, 3 different single oral samples were selected from three independent individuals (A, D and G). (**b**) Genomic variability between “clonal isolates.” In this context, 3 different clonal isolates were selected on the “sub culture” of the different strains. The whole genome of the 3 clonal isolates was analysed in order to determine the basal genomic variability between clones. In total, 3 different independent strains from our collection of *C. albicans* clinical strains were analysed (X, Y and Z). (Part of the illustration was adapted with permission from^71^).

Based on the above observation, we explored the extent of the genetic variations across isolates collected from a single carrier. Isolates A1, A2, and A3 from carrier A (all having DST 66); D1, D2, and D3 from carrier D (all having DST 1765); and G1 (DST 1768), G2, and G3 (DST 1769) from carrier G were deep-sequenced using Illumina technology (average sequencing depth, 155×, range, 110–229×; Fig. 1a and Supplementary Table S2). None of the sequenced isolates showed aneuploid chromosomes (Supplementary Fig. S1).

Single nucleotide polymorphisms (SNPs) were identified by mapping the sequencing reads to haplotype A of the *C. albicans* SC5314 reference genome^46,47^, and then inter-isolate comparisons were conducted (Table 1). Isolates from carrier A showed an average of 3830 differences. Isolates from carrier D showed an average of 757 differences. Isolates from carrier G showed an average of 1404 differences. Notably, a large majority (95.7 ± 3.1%) of the pair-wise differences was accounted by heterozygous vs homozygous genotypes. Interestingly, a similar number of differences were observed between the two isolates of identical DST obtained from carrier G (G2 vs G3, 1452 differences) than when these isolates were compared to isolate G1 that had a different DST (G1 vs G2, 1446 differences; and G3 vs G1, 1314 differences) (Table 1).

**Table 1:** Characteristics of the SNPs and LOH event detected from pair-wise genome comparison from carrier isolates (individual samples A, D and G) and clonal isolates (strains X, Y and Z).

	Material	Genome comparisons	SNP characteristics						LOH characteristics					
			Total SNPs	Heterozygous SNPs	Homozygous SNPs	Intergenic SNPs	Coding regions SNPs	Non synonymous SNPs	Number of LOH	Number of SNPs involved in LOH	Median of the maximal LOH size (MaxS) (bp)	Range of the maximal LOH size (MaxS) (bp)	Median of the minimal LOH size (MinS) (bp)	Range of the minimal LOH size (MinS) (bp)
**Single oral sample from**														
**Carrier isolate**	Individual A	A1 vs A2	2 528	2 517	11	1 144	1 384	531	291	2225	1184	21–458 739	217	2–454 851
		A2 vs A3	3 843	3 825	18	1 643	2 200	883	173	3667				
		A3 vs A1	5 119	5 082	38	2 247	2 872	1 114	297	4815				
	Individual D	D1 vs D2	767	735	32	351	416	155	111	634	738	10–339 442	57	2–91 287
		D2 vs D3	549	506	43	255	294	106	78	426				
		D3 vs D1	955	887	68	488	467	186	128	774				
	Individual G	G1 vs G2	1 446	1 361	85	694	752	282	145	1188	947	11–359 209	80	2–198 176
		G2 vs G3	1 452	1 343	109	559	893	344	145	1186				
		G3 vs G1	1 314	1 254	60	573	741	268	146	1117				
**Strain**														
**Clonal isolate**	X	X1 vs X2	395	381	14	186	229	87	66	280	354	7–96 382	19	2-2 161
		X2 vs X3	435	418	17	172	263	93	68	320				
		X3 vs X1	441	422	19	177	264	112	73	300				
	Y	Y1 vs Y2	394	390	4	136	258	92	72	276	334	17-10 099	20	2-2 722
		Y2 vs Y3	393	385	8	111	282	111	66	281				
		Y3 vs Y1	393	383	10	129	264	112	81	303				
	Z	Z1 vs Z2	352	350	2	66	286	117	60	263	262	6-78 344	18	2-2 155
		Z2 vs Z3	440	440	0	95	345	133	77	273				
		Z3 vs Z1	360	358	2	103	257	118	64	351				

(Vs = versus, for each pair-wise comparison LOH events were screened in symetric manner).

**Figure 2:**
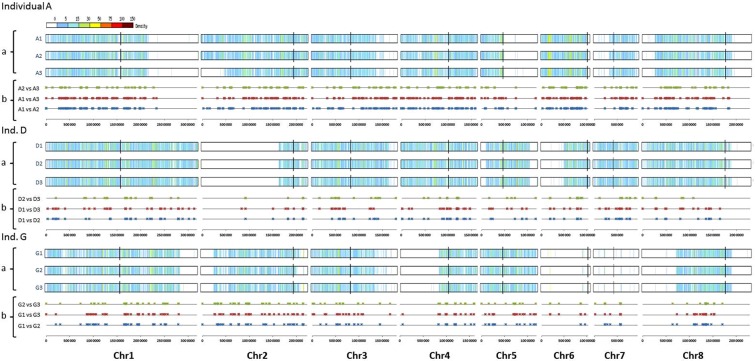
Representation of LOH events between genomes from the different carrier isolates selected from individual (Ind.) A, D and G. Panels a: Detection of large LOH event by chromosome. For each genome from isolates, heterozygous SNPs density was mapped on the 8 chromosomes (1 Kb sliding windows). Homozygous regions are indicated in light or white colour. Appearance of large LOH event (MR/BIR) between genomes is indicated by a red square. The blue vertical line indicates the centromere of each chromosome. Panels b: Density of LOH events by chromosome. For each pair of genome comparisons the starting location of all LOH events was mapped on the 8 chromosomes. For each pair-wise comparison LOH events were screened in a symetric manner. (vs = versus, Chr = chromosome).

**Figure 3:**
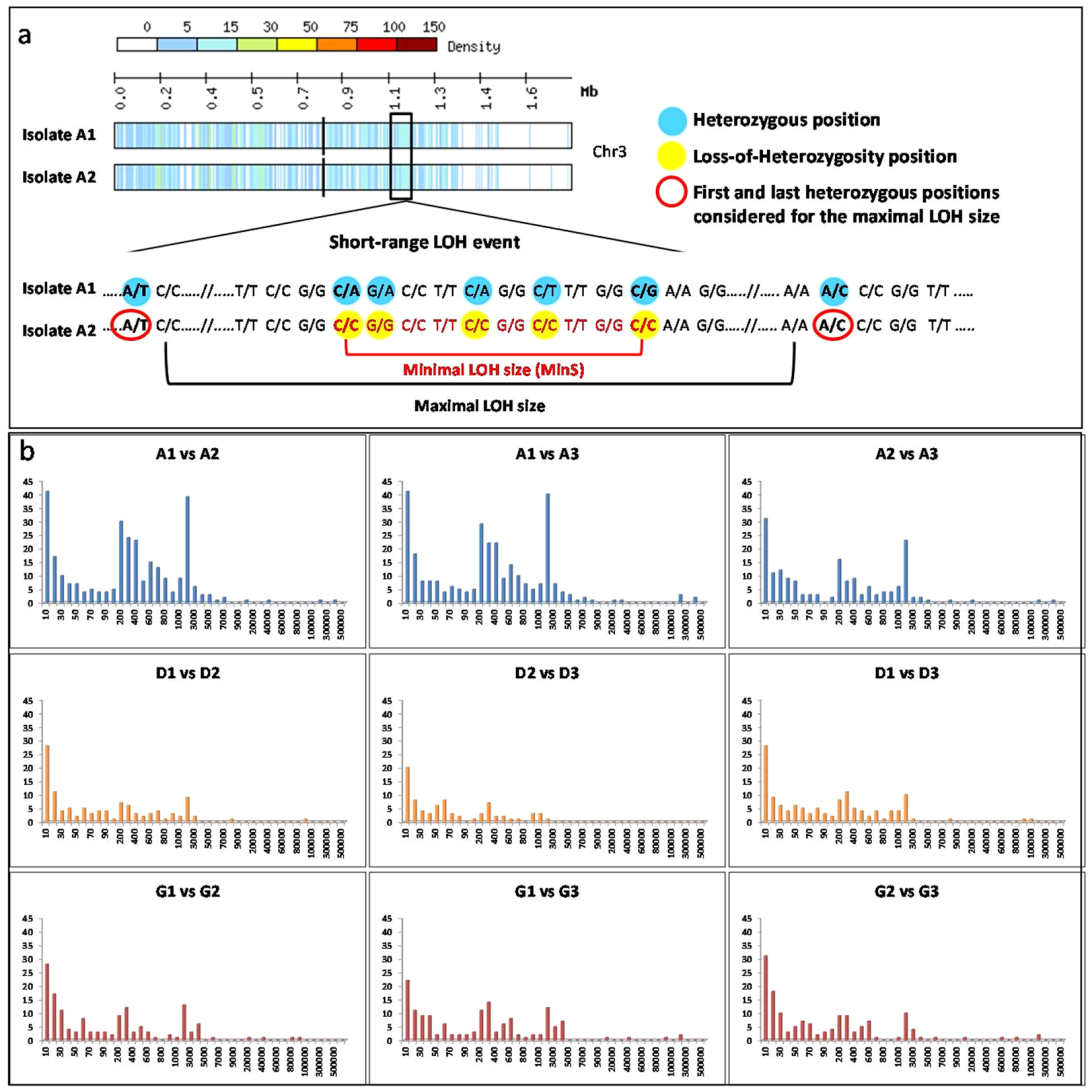
LOH characterisation (**a**). Definition of LOH event and size determination. Example of one short-range LOH event observed on chromosome 3 between the genome from carrier isolates A1 and A2. The first part represents the heatmap of the heterozygous SNPs density for the chromosome 3 of the 2 genomes (1 Kb sliding windows). The second part represents the associated diploid genome sequences. In this example the LOH MinS is 10 bps. (**b**) Distribution of MinS LOH event from the 9 pair-wise carrier isolates comparisons from individual sample A, D and G. The x-axis corresponds to the classes of LOH size (MinS in bp). The y-axis corresponds to the number of events observed by MinS classes.

**Figure 4:**
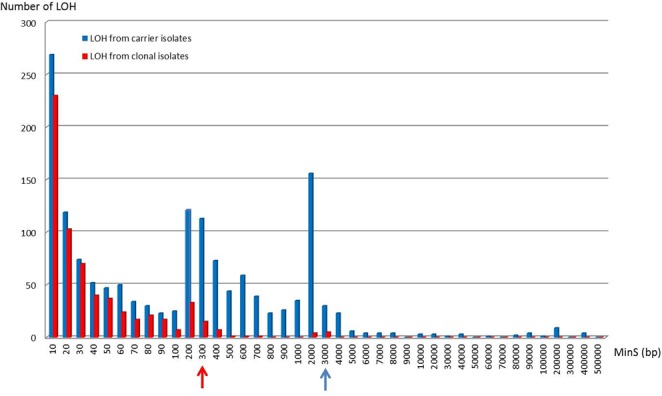
Comparison of MinS LOH event distribution between carrier isolates and Clonal isolates. The majority of LOH events (95% of the total number) identified between carrier isolates had MinS ≤ 3000 bp (blue arrow) while for clonal isolates they had a MinS ≤ 300 bp (red arrow).

**Figure 5:**
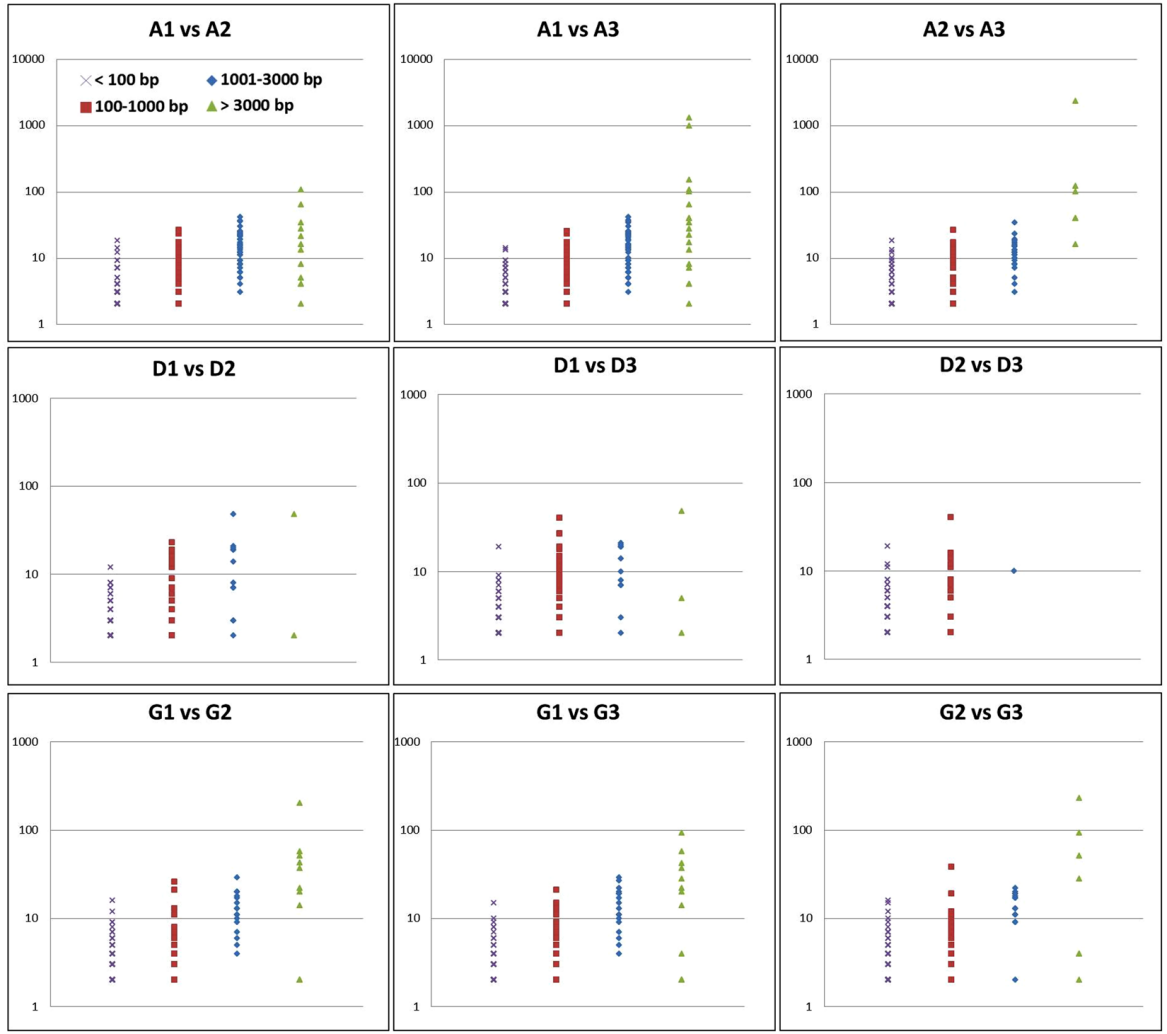
Distribution of the LOH event by MinS classes and the number of SNPs differences involved by event for the 9 pair-wise carrier isolates comparisons from individual sample A, D and G. The x-axis corresponds to the classes of LOH size (MinS in bp). The y-axis is in log scale and corresponds to the number of SNPs differences involved by LOH event. (vs = versus).

Loss-of-heterozygosity events encompassing two or more heterozygous sites have been shown to account for a large part of the genetic differences that occur between isolates from a given genetic cluster^9,12,48^. LOH events result from the loss of chromosomes (*e.g*. chromosomes 6 and 7 in the three isolates from carrier G; Fig. 2: panel a); mitotic recombination (MR) or break-induced replication (BIR) that then extend from one internal chromosomal location to the chromosomal end (*e.g*. at the right arm of chromosome 1 of all isolates from carrier A; Fig. 2: panel a); and gene conversion events that are internal to a chromosome. Importantly, we observed that most differences between isolates from the same carrier resulted from LOH events (Table 1). The number of LOH events observed between isolates varied across carriers (254, 106, and 145 on average for samples A, D, and G, respectively). We qualified the LOH events by their minimal size MinS, the distance between the two heterozygous SNPs located within the LOH and closest to its flanks (Fig. 3a and Materials and Methods). Results presented in Figs 3a and 4 showed that 95% of the LOH events had MinS values < 3 kb. Larger events were also observed (Figs 2 and 3b). For instance, isolate A3 differed from isolates A1 and A2 by a 454,851 bp MR/BIR event on the left arm of chromosome 2, and this accounted for 2271 differences between these strains (Fig. 2, panel a). Isolate G2 differed from isolates G1 and G3 by a 3538 bp MR/BIR event on the right arm of chromosome 2, and this accounted for 31 differences between these strains (Fig. 2: panel a). Additional large LOH events were detected in large, almost homozygous regions shared by the three isolates of a carrier, and thus did not account for major differences between these strains (Fig. 5).

The 5-prime ends of all LOH were mapped on the 8 chromosomes to appreciate the density of these events. As shown in Fig. 2, panels b, these LOH events were randomly distributed across heterozygous regions and affected all chromosomes. Most LOH did not extend to the chromosomal ends, suggesting that they might correspond to gene conversion events. In addition, the number of LOH differed between samples. Isolates from carrier A differed by more LOH than isolates from carriers D or G. To illustrate the complexity of these events, a complete description of the coding regions impacted by LOH events on chromosome 1 of isolates G1–G3 is presented in Supplementary Table S3. We observed alternating homozygous and heterozygous regions between the 3 genomes. In order to ensure that these LOH events were not artefacts, we performed a Sanger sequencing of 19 LOH events observed on Chr1 of isolates G1-G3. Among the 19 events tested, 18 (95%) were confirmed by Sanger sequencing.

### Within-host *C. albicans* genomic diversity was significantly higher than that of *in vitro* grown *C. albicans*

We then evaluated whether the genetic variations observed across carrier isolates could result from the propagation steps performed *in vitro* (corresponding to approx. 21–25 generations^48^) and/or our analytical pipeline. To this aim, we plated cells from three *C. albicans* strains from our collection (hereafter referred to as strains X [DST 2281], Y [DST 1047], and Z [DST 1400]) on rich medium, selected three colonies (clonal isolates) per strain (namely isolates X1-3, Y1-3, and Z1-3), prepared genomic DNA from these isolates, and subjected them to deep-sequencing using the Illumina technology (average sequencing depth, 102×; range, 58–145× (Supplementary Table S2)).

SNPs were identified as described above. Strikingly, clonal isolates obtained from any given strain differed by an average of 400 differences (range, 352–441), with a large majority (97.9 ± 1.6%) being accounted by heterozygous vs homozygous genotypes (Table 1). No large-scale genetic changes, such as aneuploidy or long-range LOH, were observed between the clonal isolates (Supplementary Figs S1 and S3). Rather, all pair-wise comparisons revealed short-range LOH (average number, 70; range, 60–81) whose MinS distribution was displaced towards smaller sizes compared to that observed for isolates obtained from carriers (Fig. 4 and Supplementary Fig. S2). Similar to those observed in isolates from healthy carriers, these LOH events were randomly distributed across heterozygous regions and affected all chromosomes (Supplementary Fig. S3: panels b).

**Figure 6:**
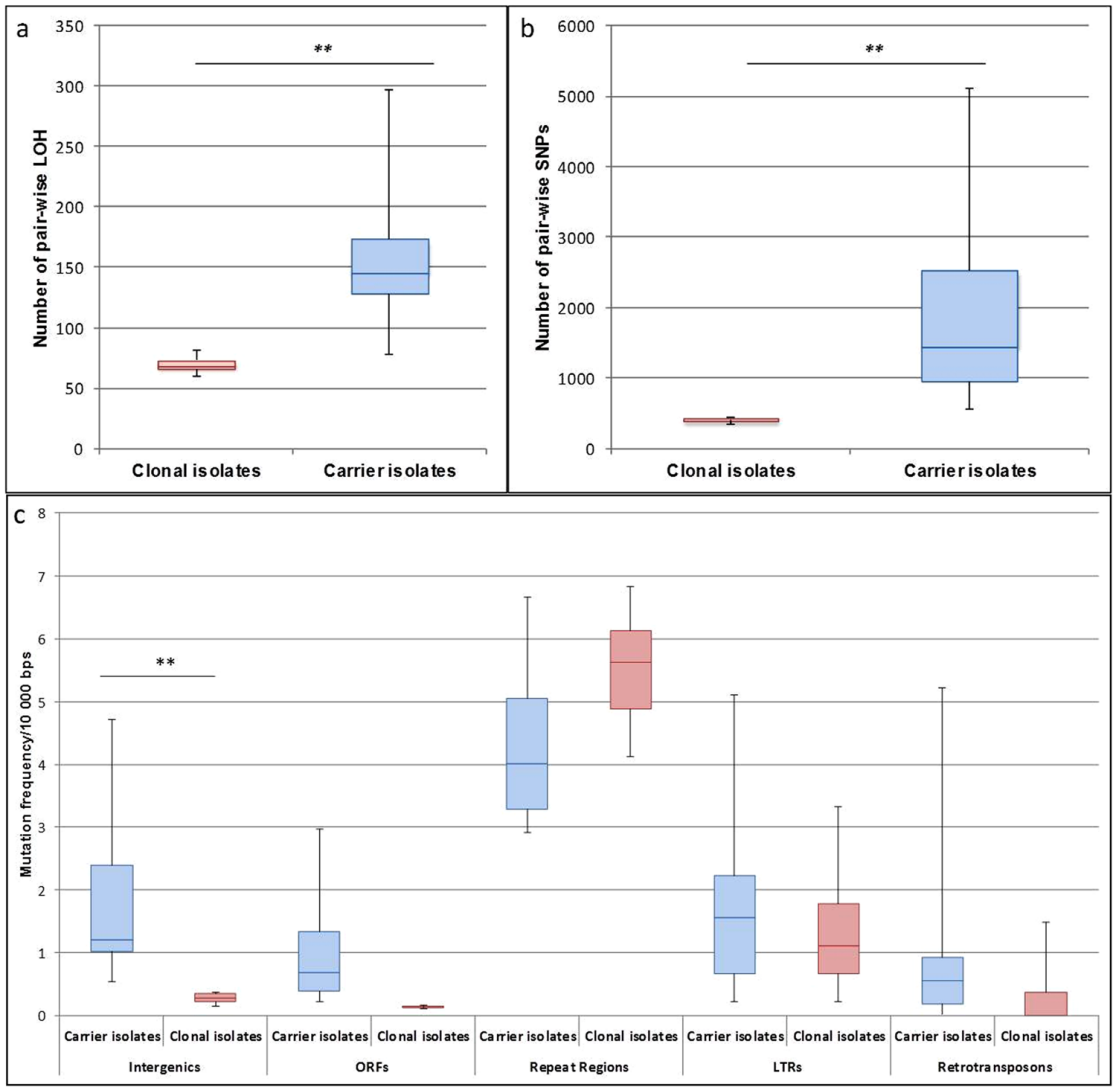
Statistical analysis of the genomic diversity between carrier and clonal isolates. (**a**) Comparison of the number of LOH events observed from genomes of clonal and carrier isolates. The number of LOH events observed between the genomes from carrier isolates was significantly higher than thus observed between the genome from clonal isolates (One way ANOVA test, p = 0.0013). (**b**) Comparison of the number of SNPs between genomes from clonal and carrier isolates. The number of SNPs detected between the genomes from carrier isolates was significantly higher than thus observed between the genome from clonal isolates (One way ANOVA test, p = 0.007). (**c**)Mutation frequency comparisons for carrier and clonal isolates by genomic regions. The mutation frequencies were represented for 10.000 bps of the different genomic regions. Repeat regions were significantly more mutated than other features whatever group considered (carrier or clonal isolates) (**p < 0.01; Post-hoc test: Tukey HSD). Carrier isolates were significantly more variable in intergenic regions than clonal isolates (**p < 0.01, Post-hoc test: Tukey HSD).

The genomic variability between the carrier isolates (A1-3, D1-3, and G1-3) or clonal isolates (X1-3, Y1-3, and Z1-3) was quantified using the numbers of pair-wise SNPs and pair-wise LOH events. For each criterion, the diversity of the carrier isolates was significantly higher than the diversity of clonal isolates (p = 0.007 for SNPs, and p = 0.0013 for LOH events; one way ANOVA test) (Fig. 6a,b). By focusing on SNPs, we studied the pair-wise variability (mutation frequency) across different types of genomic features that have been defined in the *C. albicans* genome, namely intergenic regions, ORFs, repeat regions, long-terminal repeats (LTRs), and retrotransposons. A two-way ANOVA showed a significant difference between carrier isolates and clonal isolates and between genomic features. Indeed, repeat regions were significantly more mutated than other features of whatever group that was considered (carrier or clonal isolates; p < 0.01; post-hoc test: Tukey honestly significant difference [HSD]). Furthermore, carrier isolates were significantly more variable in intergenic regions than clonal isolates (p < 0.01, post-hoc test: Tukey HSD) (Fig. 6c).

**Figure 7:**
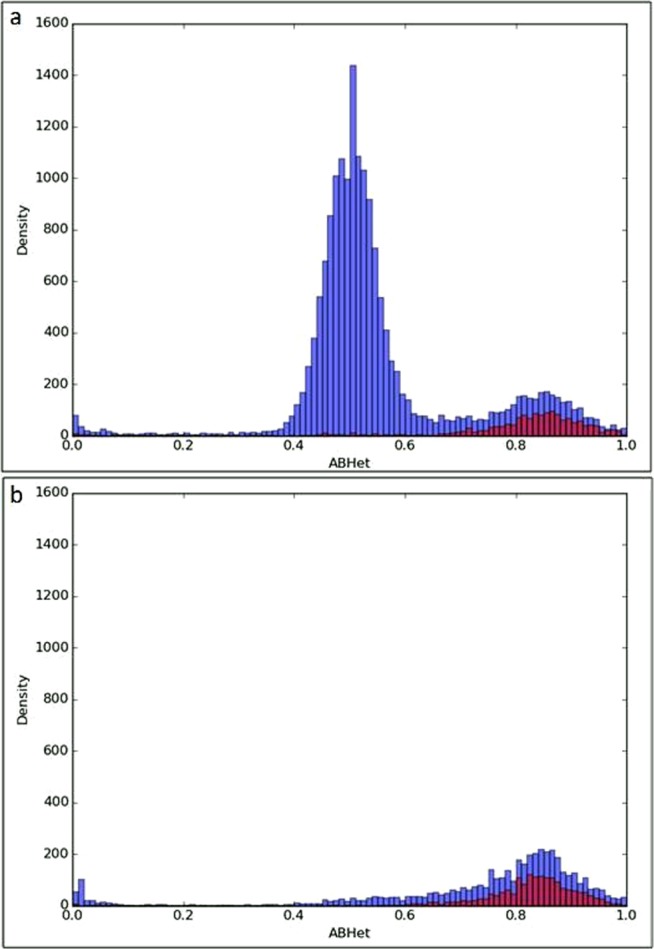
Comparison of the allelic ratio distribution of all heterozygous polymorphic positions. (**a**) Comparison from carrier isolates. (**b**) Comparison from clonal isolates. The allelic ratio (ABHet = number of reads for reference allele / total number of reads) was determined with GATK tools and histograms were built based on the number of SNPs with AbHet values in a given interval (bin = 0.02). For each heterozygous SNP differences between pair of genomes the ABHet ratio was plot on the histogram. Red color represents the ABHet ratios from the positions located in the repeated regions, retrotransposons and LTRs and blue color the positions located in the other regions of the genome. For carrier isolates (**a**), we observed a bimodal distribution with the majority achieving a Gaussian distribution centred on a ABHet value of 0.5, which is the expected value for a diploid genome, and a minority achieving a Gaussian distribution centred on a ABHet value of 0.85, which is the limit cut-off for the heterozygous SNPs definition in our pipeline. This last distribution (around 0.85) was the only one found for clonal isolates (**b**).

Because LOH events in clonal isolates were significantly smaller than those observed in carrier isolates (Fig. 4), we evaluated whether they could in part result from miscalling of polymorphic positions. Toward this aim, we inspected the allelic ratio (ABHet = number of reads for reference allele/total number of reads) of all polymorphic positions, with the expectation that they should have a Gaussian distribution centred on a ABHet value of 0.5 for a diploid genome. The results presented in Fig. 7a showed that ABHet values obtained for carrier isolates had a bimodal distribution with the majority achieving a Gaussian distribution centred on a ABHet value of 0.5 and a minority achieving a Gaussian distribution centred on a ABHet value of 0.85. In contrast, ABHet values obtained for clonal isolates had a Gaussian distribution centred on a value around 0.85 (Fig. 7b). To further investigate the second modal distribution centered around 0.85, we associated the location of SNPs on the genome and their ABHet ratios. We found that a large part of the SNPs associated with ABHet value around 0.85 were located in repeated regions of the genome, including retrotransposons, LTRs and repeat regions. (Fig. 7). These SNPs are partly excluded in our analytical pipeline that only considers polymorphisms with ABHet values > 0.15 and <0.85 as heterozygous. Accordingly, some of the SNPs located in repeated regions are not excluded and may cause artefacts in the identification of LOH. It may ensue that most LOH events identified in pair-wise comparison of clonal isolates are artefactual, corresponding partly to repeat regions (Fig. 7b) and explaining their smaller size compared to those observed in pair-wise comparisons of carrier isolates (Fig. 4). Thus, the number of LOH events in clonal isolates might be overestimated (as well the number of LOH events in carrier isolates that also show a population of LOH events similar in size to those in the clonal isolates (Fig. 4)), thus reinforcing our conclusion that the level of genomic diversity observed between the *C. albicans* isolates obtained from oral carriers was significantly higher than that observed between clonal isolates obtained *in vitro*. Taken together, our results showed that a single oral sample exhibits a genetically heterogeneous *C. albicans* cell population.

## Discussion

This work represents, to our knowledge, the first genome-wide analysis of the within-host diversity of *C. albicans* in healthy individuals. We first explored the level of oral *Candida* carriage in a group of healthy young individuals. As expected, the carriage of *C. albicans* was common in this population with a prevalence of 17.9%, while other *Candida* species were rarely found, emphasizing the predominant role of *C. albicans* in the normal oral microbiota of humans^4,39^. We then studied the genomic diversity of *C. albicans* within each healthy carrier. To this aim, we analysed several isolates from oral samples and determined the intrinsic level of genomic diversity in the context of commensalism. Overall, our results showed that a single oral sample exhibited a genetically heterogeneous *C. albicans* population, with isolates differing from each other by multiple short-range LOH events corresponding to gene conversion tracts. MLST is insufficient to pinpoint this heterogeneity, except in rare cases where a short-range LOH or SNP affected one of the MLST loci, as was the case for only one isolate in our study.

Whole genome sequencing offers opportunities to extend the analysis of *C. albicans* diversity at the genomic level^36,49^. Unlike longitudinal studies evaluating the evolution of the *C. albicans* genome in patients using only one colony per sample at different time points^36,50^, our analysis characterised several isolates from the same oral sample collected at a single time point. For each sample, we compared the whole genome of 3 different isolates, focusing on pair-wise SNPs and LOH events. Indeed, the occurrence of these events has been correlated with the response of *C. albicans* to various stresses both *in vitro* and *in vivo*^30,31,33,51–53^. However, little is known about the occurrence and the role of these events during commensalism in humans^54^.

As mentioned above, while a low level of genotypic diversity was found by MLST, the genome-wide analysis identified numerous genetic variants, mainly short-range LOH events, between carrier isolates, indicating a genetically heterogeneous *C. albicans* population in oral samples of healthy individuals. To confirm that these events were not due to sequencing errors, to our genome analysis pipeline, or to clonal reproduction *in vitro*, we quantified and compared the extent of variations observed between carrier isolates and those observed between clonal isolates. Our results showed that the level of genomic diversity observed between the *C. albicans* isolates obtained from oral carriers was significantly higher than that observed between clonal isolates propagated *in vitro*, confirming that oral samples harbour a genetically heterogeneous *C. albicans* population. The diversity across carrier isolates may reflect the evolutionary consequences of life-long commensal carriage, initiated through mother-to-child transmission, and the recurrent exposure of individual isolates to genotoxic stress, such as upon interaction with immune cells or environmental changes. The occurrence of such events was already reported *in vitro* under stress conditions. For instance, *in vitro* exposure of *C. albicans* cells to oxidative stress (such as during phagocytosis) caused an increased level of short-tract LOH events resulting from recombinations^31^. Such events were also more frequent in mouse models of oral or systemic *C. albicans* infection than upon *in vitro* growth^33,35^. Differences in the extent of divergence between isolates from a single carrier (*e.g*. isolates from carrier A were more divergent between each other than isolates from carrier B or G) may reflect a longer divergence time or differences in terms of genomic plasticity. Importantly, genomic differences across carrier isolates were mostly accounted by short-range (gene conversion) LOH events. No example of aneuploidy was observed across the nine investigated carrier isolates (Fig. S1), and only a few long-range BIR/MC events were identified. Interestingly, while this paper was under evaluation, Ene *et al*. published a study evaluating mutations arising during passaging *in vitro* and in mouse models of gastrointestinal colonisation^48^. Similarly, they showed that microevolution was primarily driven by de novo base substitutions and frequent short-tract LOH events while large-scale chromosomal changes were rare. Our results, performed in humans, are consistent with the observations made by Ene *et al*. and confirmed the relevance of what they observed *in vivo*^48^.

Additionally, a direct comparison of the mutation frequency across the different types of genomic features was performed between carrier and clonal isolates. The carrier isolates exhibited a significantly higher number of SNPs in intergenic regions as compared to clonal isolates. (Fig. 6c). This was in contrast with repeat regions, LTRs, and retrotransposons that showed similar mutation frequencies between carrier and clonal isolates. Hence, SNPs and LOH events distinguish isolates within a carrier, because their enrichment in intergenic regions and ORFs may have functional consequences. Of note, we observed a high frequency of SNPs in repeat regions for both carrier and clonal isolates. Elevated mutation rates associated with repetitive regions of the *C. albicans* genome were reported several times and reflects the limitations of SNP analyses within structurally complex regions^48,55–57^. Our results suggest that *C. albicans* diversity in the healthy host might be higher than could have been anticipated from studies that have used MLST. This probably reflects the fact that most epidemiological studies have typed one isolate per individual and therefore did not address intra-host variability. More importantly, it reflects the higher discriminatory power of whole genome sequencing over MLST. We suppose that diversifying lineages coexist in each human individual, and that this diversity could enable rapid adaptation under stress exposure. Indeed, the human digestive tract is the main reservoir for infection from which the most suitable variant could emerge^7,8^. It is known that *C. albicans* genomic variations, ranging from SNPs to large scale genetic changes, can facilitate adaptation to environmental changes and improve the persistence of the fungus in various host niches^58^. The main example is the link to genomic variations in *C. albicans* isolates that were resistant to antifungal drugs^51,59^. However, other studies have shown the implication of genetic variations on *C. albicans* pathogenicity by modifying the expression of virulence factors and the interaction with the host^9,57,60–63^.

The natural diversity observed during commensalism might explain the remarkable ability of *C. albicans* to adapt to stress conditions within the host as well as the disparate response observed during infections. Our results provide crucial information for future longitudinal studies aimed at evaluating genomic evolution in healthy individuals or patients. Indeed, these studies offer a unique view at the *in viv*o evolution of *C. albicans*^36^. For instance, work by Ford *et al*. (2015) has revealed the occurrence of numerous genomic changes over time in *C. albicans* strains isolated from patients throughout the course of a fluconazole treatment^36^. However, in this study, only one isolate was analysed at each time point, and it cannot be excluded that some of the variations observed between isolates may have pre-existed due to intrinsic diversity of *C. albicans* in the healthy host. Hence, future longitudinal studies should, whenever possible, assess multiple isolates at each time point in order to disentangle pre-existing variations from those acquired in the course of the study.

## Material and Methods

### Study cohort and sample collection

The studied population comprised of 56 20-to-22-year-old healthy volunteers from a French military school. The students were living across 2 different dormitories, one for members of the swim team and the other for members of the volleyball team. Both groups shared the same hygiene facilities and canteen, and none of them has taken antibiotics within 6 months. They also attended common classes and military preparation. The general information had been given collectively during classes by one of the investigators. The informed consent was obtained from all student participants.

Swabs were extemporaneously plated on a chromogenic plate BBL™ CHROMagar™ *Candida* (BioMérieux, Marcy-l’étoile, France) and incubated for 5 days at 37 °C. Colonies from each plate were enumerated. *C. albicans* detection was based on growth colour on CHROMagar™ plate typically represented in green. Up to 8 green single colonies (presumptive *C. albicans* strains) were picked and sub-cultured onto separate media for species identification by mass spectrometry-type Maldi-TOF. For storage, each single colony recovered from the plate were grown overnight at 30 °C in liquid YPD medium before being frozen in 30% glycerol at −80 °C. For the next analysis, the yeast cells were grown at 30 °C in liquid or solid media in YPD (1% yeast extract, 2% peptone, 2% dextrose). Solid media were obtained by adding 2% of agar.

### Multilocus Sequence Typing

*C. albicans* isolates obtained from primo culture of oral swabbing of each healthy carrier were typed using MLST as described previously^64^. Briefly, alleles of the seven housekeeping genes (*AAT1a, ACC1, ADP1, MPIb, SYA1, VPS13*, and *ZWF1b*) were amplified and sequenced for each isolate. Sequencing was performed on both strands using an ABI Prism 3130xl genetic analyzer (Applied Biosystems, Foster City, CA, USA). Allele and diploid sequence type (DST) assignments were determined using the *C. albicans* multilocus sequence typing database (http://pubmlst.org/calbicans).

### Illumina sequencing

To extract genomic DNA, 2 µl of stored solution was cultured on a YPD plate, for 48 h at 30 °C. One single clonal colony was then picked-up and grown overnight at 30 °C in 50 ml of liquid YPD. The DNA extraction was performed using the QIAamp® DNA Mini Kit (Qiagen, Courtaboeuf, France), according to the manufacturer’s instruction, with an additional mechanical lysis step (FastPrep; MP Biomedicals, Illkirch, France) following the addition of chemical lysis buffer. Genomic DNA was processed to prepare libraries for Illumina sequencing. Libraries were prepared using the NEXTflex™ PCR-Free DNA Sequencing kit (Illumina®) according to the manufacturer’s recommendations. HiSeq2000 or HiSeq2500 platforms was used to generate 101 bp paired-ends reads.

### Genomic variants analysis

Sequences and genomic variations were analysed as previously described by Ropars *et al*.^12^. Briefly, sequences were mapped to the genome of *C. albicans* reference strain SC5314, assembly 22 (version A22-s06-m01-r01), available from CGD^65^ using BWA v0.7.7 with default parameters^66^. The next processing was performed with the Genome analysis toolkit v3.1^67^. To minimize false-positive SNP calls near insertion/deletion events, poorly aligning regions were identified and realigned using the GATK RealignerTargetCreator and IndelRealigner modules. The variant calling was performed with a GATK HaplotypeCaller. Poor quality SNPs were filtered using the GATK VariantFiltration module, with best practices recommended annotation for hard filters (QD < 2.0, MQ < 40.0, FS > 60.0, HaplotypeScore >13.0, MQRankSum <−12.5, ReadPosRankSum <−8.0). Then we filtered.vcf files to select SNPs with a minimal sequencing depth of 18×. We evaluated the allelic balance for heterozygous calls (ABHet) and homozygous calls (ABHom) with AlleleBalance annotation GATK module (Tables S4 and S5). Heterozygous SNPs were defined as positions where 15% or more of the calls showed one allele, and 85% or less of the calls showed a second allele. Homozygous SNPs were defined as positions where more than 98% of the calls differed from the reference genome^12,68^. The output files gather high quality SNPs used to compare each pair of genomes from isolates or clonal colonies and a complete LOH analysis report (number of event, minimal and maximal size). LOH events were defined by at least 2 successive losses of heterozygous SNP positions (transition from a heterozygous to homozygous position between two genome comparisons). These events were analysed in a symetric manner *e.g*. A1 to A2 and A2 to A1. The minimal LOH event size (MinS) was defined by the distance between the two heterozygous positions located within the LOH event and closest to its flanks. The maximal LOH event size (MaxS) was defined by the number of base pairs between the heterozygous positions found before and after the LOH event observed on the genome (Fig. 3a). At the end of the process, we generated figures summarizing all of the data, such as sequencing depth for the identification of aneuploidies and heterozygosity density maps (performed for 1 or 10 kb sliding windows) plotted across the 8 chromosomes as described previously^69,70^.

### Sanger sequencing

Selections of ORFs impacted by a LOH event across chromosome 1 of isolates from individual G (G1, G2, and G3) were confirmed by Sanger sequencing. PCR was performed in an Eppendorf Mastercycler ep gradient thermal cycler. The PCR mixture contained 1 µl of the extracted genomic DNA, 2 µl of the 10 × PCR buffer; 2 µl MgCl_2_ (50 mM); 1 µl of a mix of deoxynucleoside triphosphates (dNTP) (5 mM); 0.5 µl (each) primer (10 µM); 0.2 µl of Taq polymerase (Invitrogen), and water to a final volume of 20 µl. Primers used in this study are listed in Supplementary Table S6. The following conditions were used: initial denaturation at 94 °C for 3 min; 30 cycles with denaturation at 94 °C for 40 s, annealing at 54 °C for 40 s, and extension at 72 °C for 1 min/kb; and a final extension time at 72 °C for 10 min. The PCR products were verified by electrophoresis on a 1% agarose gel. The PCR products were then sequenced at Eurofins sequencing facility using ABI 3730XL sequencing machines (Applied Biosystems). Sequence analysis and SNP detection were performed using the SeqScape™ v3.0 software.

### Statistical analysis

Statistical significance was determined using one way ANOVA (Fig. 6a,b), and two-way ANOVA with repeated measures and post-hoc Tukey HSD tests (Fig. 6c). A p < 0.01 was considered significant and denoted by a double asterisk.

### Ethics Statement

The study was performed in accordance with the Declaration of Helsinki and the best National Recommendations at the moment of the sampling. The study was approved by the training committee of the Ecole Polytechnique France 20112008. The informed consent was obtained from all student participants.

## Supplementary information

Supplementary tables and figures

Table S4

Table S5

## Data Availability

All code and relevant datasets generated during and/or analysed during the current study are available from the corresponding author upon request. The dedicated script allowing the analysis of LOH has been submitted on the github (https://github.com/maufrais/WHGDCA_ES). Raw reads have been deposited at the NCBI Sequence Read Archive under BioProject ID PRJNA489773 [https://www.ncbi.nlm.nih.govbioproject/489773].
