# Supplementary material for: Within-Host Genomic Diversity of Candida albicans in Healthy Carriers
Source: Sci Rep. 2019 Feb 22;9:2563. doi: 10.1038/s41598-019-38768-4 (PMC6385308; doi:10.1038/s41598-019-38768-4)
Supplement: Supplementary file 1 — Supplementary tables and figures [file 41598_2019_38768_MOESM1_ESM.pdf]

Within-Host Genomic Diversity of *Candida albicans* in Healthy Carriers

Emilie Sitterlé[1,2,3], Corinne Maufrais[4], Natacha Sertour[1], Matthieu Palayret[5], Christophe d'Enfert[1], Marie-Elisabeth Bougnoux[1,3*]

Table S1: **Principal characteristic of the *C. albicans* isolates obtained from the oral primo culture of the 10 healthy carriers.**

			Isolate		MLST									
Individual	Age	Sex	Number of *C. albicans* isolate in primo culture	Name of isolate	*ACC1*	*AAT1*	*ADP1*	*MPIb*	*SAY1*	*VPS13*	*ZWF1b*		DST	Clade
A	21	M	8	A1-8	3	2	5	2	2	6	5		66	1
B	21	M	2	B1-2	3	2	5	2	2	24	5		417	1
C	20	M	1	C1	14	43	6	4	7	3	8		1771	4
D	22	M	16	D1-8	4	36	4	94	4	26	4		1765	2
E	21	M	8	E1-8	3	8	6	4	7	3	35		1766	4
F	20	M	19	F1-8	3	3	10	3	26	39	95		1767	9
G	21	M	10	G1	7	13	15	60	34	37	12	ATTTTCCATTA	1768	3
				G2-8	7	13	15	60	34	37	15	MYTYYCCAYTA	1769	3
H	22	M	4	H1-4	5	2	5	2	2	6	5		69	1
I	22	M	1	I1	5	2	5	2	2	6	5		69	1
J	20	F	1	J1	7	4	25	41	160	118	6		1770	3

Table S2. MLST and sequencing summary data from carrier or clonal isolates.

		MLST		Illumina	
		Clade	DST	Reads	Sequencing depth (mean)
**Single oral sample from**	**Carrier isolate**				
Individual A	A1	1	66	8 709 970	123
	A4	1	66	12 498 872	176
	A8	1	66	7 973 129	112
Individual D	D2	2	1765	12 430 900	172
	D4	2	1765	10 394 399	144
	D6	2	1765	10 326 576	144
Individual G	G1	3	1768	11 762 371	187
	G2	3	1769	8 684 792	110
	G3	3	1769	14 439 056	229
**Strain**	**Clonal isolate**				
X	X1	4	2281	8 954 225	98
	X2	4	2281	8 048 372	92
	X3	4	2281	7 396 486	86
Y	Y1	1	1047	4 600 098	58
	Y2	1	1047	10 436 389	130
	Y3	1	1047	8 913 126	110
Z	Z1	1	1400	11 408 364	145
	Z2	1	1400	7 740 394	97
	Z3	1	1400	8 232 147	103

Table S3: Cartography of coding regions impacted by LOH events on chromosome 1 for the 3 genomes analysed for the individual G. Grey boxes indicate heterozygous regions and white boxes homozygous regions.

Chromosome 1								
LOH position start	LOH position end	LOH size (MinS in bp)	ORF name	G1	G2	G3	Confirmation by sanger sequencing	Number of LOH positions analyzed on the fragment
207292	209765	2473	C1_01050C_A<br>MBF1<br>C1_01070C_A		grey	grey	Yes	8
296151	297134	983	C1_01500W_A		grey		not tested	
723 588	728071	4483	C1_03450C_A<br>C1_03460C_A	grey		grey	yes	3
867415	867976	561	IFD6	grey			not tested	
925378	926429	1051	TUS1		grey	grey	Yes	6
955818	957058	1240	C1_04630C_A		grey		Yes	4
982992	984515	1523	LYS21		grey	grey	not tested	
1021162	1021631	469	C1_04920C_A		grey		not tested	
1053374	1055986	2612	ARG2<br>PSF2<br>RRP6		grey	grey	NO	9
1109047	1110523	1476	C1_05280W_A	grey		grey	Yes	4
1203093	1204760	1667	C1_05750C_A<br>PGA26	grey	grey		Yes	6
1255766	1255989	223	C1_05970W_A	grey			not tested	
1256172	1256412	240	C1_05970W_A	grey			not tested	
1278773	1280984	2211	MSI3		grey	grey	Yes	5
1658913	1659854	941	C1_07650W_A		grey	grey	not tested	
1680887	1681713	826	C1_07730W_A	grey		grey	not tested	
1684233	1685604	1371	C1_07730W_A		grey			
1685983	1686115	132	C1_07730W_A			grey		
1689714	1690340	626	C1_07730W_A		grey			
1703162	1706234	3072	C1_07810C_A<br>C1_07820W_A	grey	grey		Yes	7
1736705	1737306	601	C1_07980C_A		grey	grey	not tested	
1791007	1791449	442	CAT8		grey	grey	Yes	5
1796848	1800880	4032	MLT1		grey		not tested	
1925226	1926447	1221	AFG1<br>C1_08860C_A	grey		grey	Yes	8
1931633	1934096	2463	HEM14			grey	Yes	5
1942700	1949589	6889	PFK26	grey		grey	not tested	
1951516	1956207	4691	PFK26<br>C1_08970W_A<br>ZWF1		grey	grey	Yes	4
1958916	1960411	1495	KEX2<br>C1_09000W_A		grey	grey	not tested	
1972754	1973040	286	C1_09060C_A	grey			not tested	
2170989	2172468	1479	VPS41	grey	grey		not tested	
2174039	2174769	730	C1_09940W_A<br>C1_09950C_A	grey	grey		Yes	10
2259086	2262213	3127	C1_10230C_A		grey	grey	Yes	4
2272828	2273967	1139	GCA1		grey	grey	not tested	
2425525	2426803	1278	RTG3<br>C1_11000C_A		grey	grey	not tested	
2475466	2477757	2291	C1_11270W_A<br>C1_11280W_A	grey	grey		Yes	4
2477830	2481511	3681	C1_11290W_A<br>PSP1	grey	grey		Yes	4
2513020	2513962	942	SAM2		grey		not tested	
2515127	2519573	4446	C1_11460W_A<br>DPP1		grey	grey	Yes	5
2542170	2544832	2662	PLD1	grey		grey	Yes	7

Table S4: Characteristics variant quality parameters and ABHet values for polymorphism positions detected between carrier isolates. (QUAL= quality, QD= quality by depth, DP= depth of coverage, MQ= mapping quality, ABHet = reference allelic ratio, GT= genotype and GQ= genotype quality). (See Supplementary dataset Table S4)

Table S5: Characteristics variant quality parameters and ABHet values for polymorphism positions detected between clonal isolates. (QUAL= quality, QD= quality by depth, DP= depth of coverage, MQ= mapping quality, ABHet = reference allelic ratio, GT= genotype and GQ= genotype quality). (See Supplementary dataset Table S5).

Table S6: Primers used to confirm LOH events.

Primer name	Sequence
Ca22chr1A_208432-209275_F	AACTGCCAACACCAAGTC
Ca22chr1A_208432-209275_R	CGGTAGAGCAACAACAAT
Ca22chr1A_727151-727560_F	TGTTGGCGGGAAAACATT
Ca22chr1A_727151-727560_R	CCAGCATGTGCCTCTTCA
Ca22chr1A_925531-926239_F	TGCTTTGGACCTTTCGGC
Ca22chr1A_925531-926239_R	AAAAAACTACCGAACTCC
Ca22chr1A_ 955982-956386_F	CAAATCCTTCTTCTTTTG
Ca22chr1A_ 955982-956386_R	TATTATCGTTGGTATTGG
Ca22chr1A_1019822-1020221_F	GATACCAAATCATGTGAA
Ca22chr1A_1019822-1020221_R	TGATTCAAGTTATTCTGA
Ca22chr1A_1054078-1054870_F	TTGTCACGTATCTATTCT
Ca22chr1A_1054078-1054870_R	TAGACTACTACTATCTAG
Ca22chr1A_1109780-1110453_F	CTCTGGAGTAGGGTCAAT
Ca22chr1A_1109780-1110453_R	CTCAATTGATTTACCAAC
Ca22chr1A_1203706-1204259_F	CCCTCATTATTGATTAGT
Ca22chr1A_1203706-1204259_R	TACCAAGCAGTCTTTCCC
Ca22chr1A_1280077-1280540_F	ACATCAACAAAGGCAACT
Ca22chr1A_1280077-1280540_R	GGTGAAAGTGCTAAGAAT
Ca22chr1A_1705477-1706195 _F	CTGGTGTTATGAGTGGAT
Ca22chr1A_1705477-1706195 _R	TTAAAAATCTTGGGAAGG
Ca22chr1A_1791150-1791539_F	TTGACGACGAGGTTGATG
Ca22chr1A_1791150-1791539_R	AACATTCATAAGAAAGCG
Ca22chr1A_1926025-1926508_F	AACTTGCATTGTGAGTTT
Ca22chr1A_1926025-1926508_R	CAGAGTTTTTGAATAAACTG
Ca22chr1A_1931660-1932134_F	TTCTTCAGGGGAAATCAT
Ca22chr1A_1931660-1932134_R	TAAGGTTTCCATTCCATC
Ca22chr1A_ZWF1_F	GTTTCATTTGATCCTGAAGC
Ca22chr1A_ZWF1_R	GCCATTGATAAGTACCTGGAT
Ca22chr1A_2171026-2171696_F	CACACACACCCACACACA
Ca22chr1A_2171026-2171696_R	TACTGGTAGCGTTTTTAA
Ca22chr1A_2174288-2174676_F	ATCCAACTAATAATCGTC
Ca22chr1A_2174288-2174676_R	AATTGAACCAGATGAACC
Ca22chr1A_2259122-2259818_F	AACATACATATACAGATC
Ca22chr1A_2259122-2259818_R	GTACTCTTTGGCAAAGGG
Ca22chr1A_2321844-2322256_F	GTTGAAATATCTTTCACC
Ca22chr1A_2321844-2322256_R	CGAAAACTATTTTTTCAGC
Ca22chr1A_2476789-2477561_F	CAATATAACATATGGTATTCC
Ca22chr1A_2476789-2477561_R	TGAAAGAGAATTGACCCT
Ca22chr1A_2478121-2478546_F	TCATTATGGATCATTTGC
Ca22chr1A_2478121-2478546_R	TCCATTAGCACAATGTAT
Ca22chr1A_2517400-2518253_F	CTACCAAAAACAAATAATCTC
Ca22chr1A_2517400-2518253_R	CTATTCCACCAAATCCTC
Ca22chr1A_2542450-2542980_F	TCGTGACGACTCATCACA
Ca22chr1A_2542450-2542980_R	ATTGCAGGAGCACCAGTG

Figure S1. Representation of the sequencing depth obtained for the 9 carrier isolates.
The average normalized sequencing depth on the y-axis (sliding windows of 10 kb) is relative to diploid levels of the genome and plotted across the 8 chromosomes.

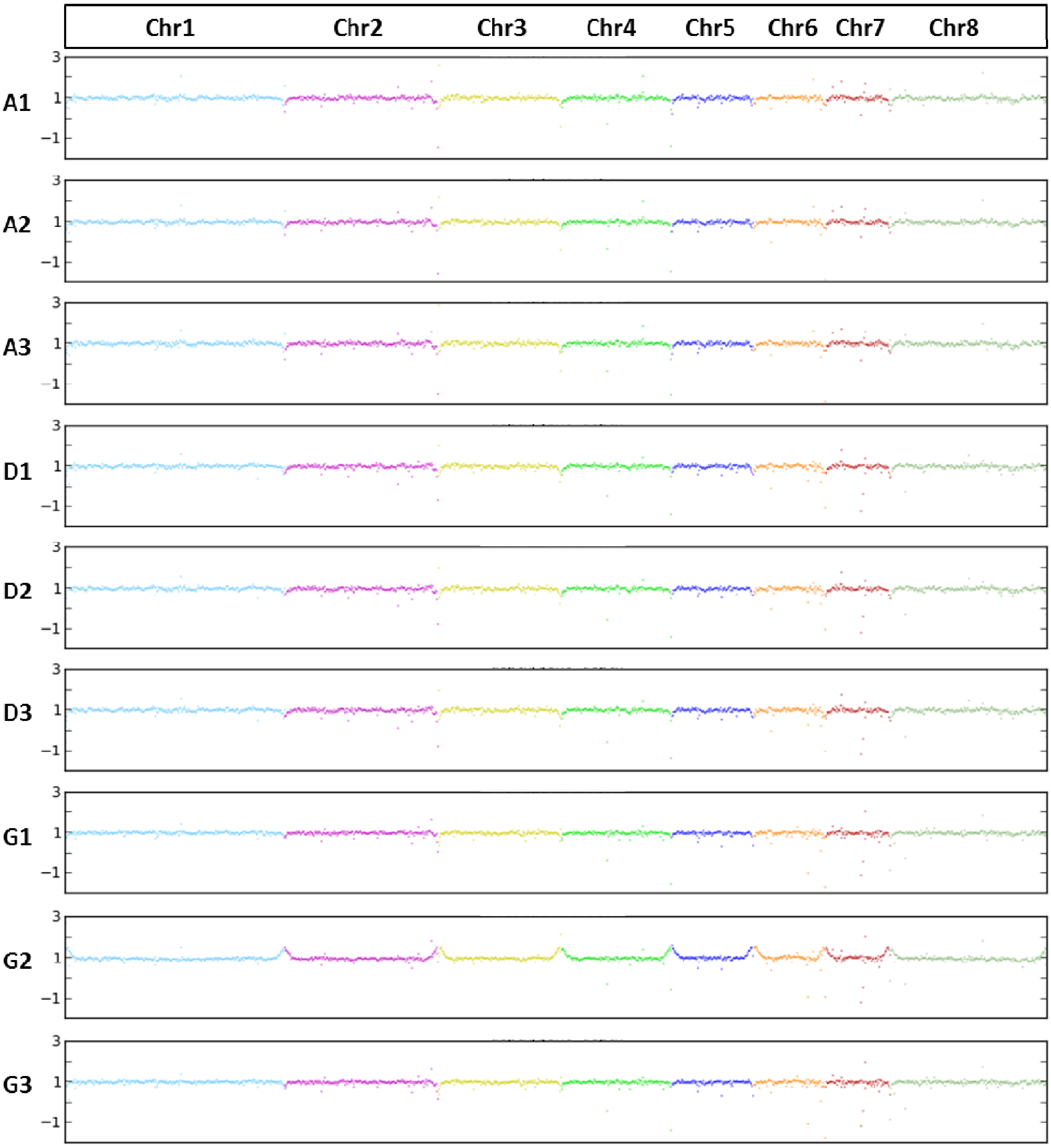

Figure S2. Distribution of MinS LOH events from the 9 pair-wise clonal isolates comparisons from strains X, Y and Z. The x-axis corresponds to the class of LOH size (MinS in bp). The y-axis corresponds to the number of events observed by MinS classes. (vs = versus)

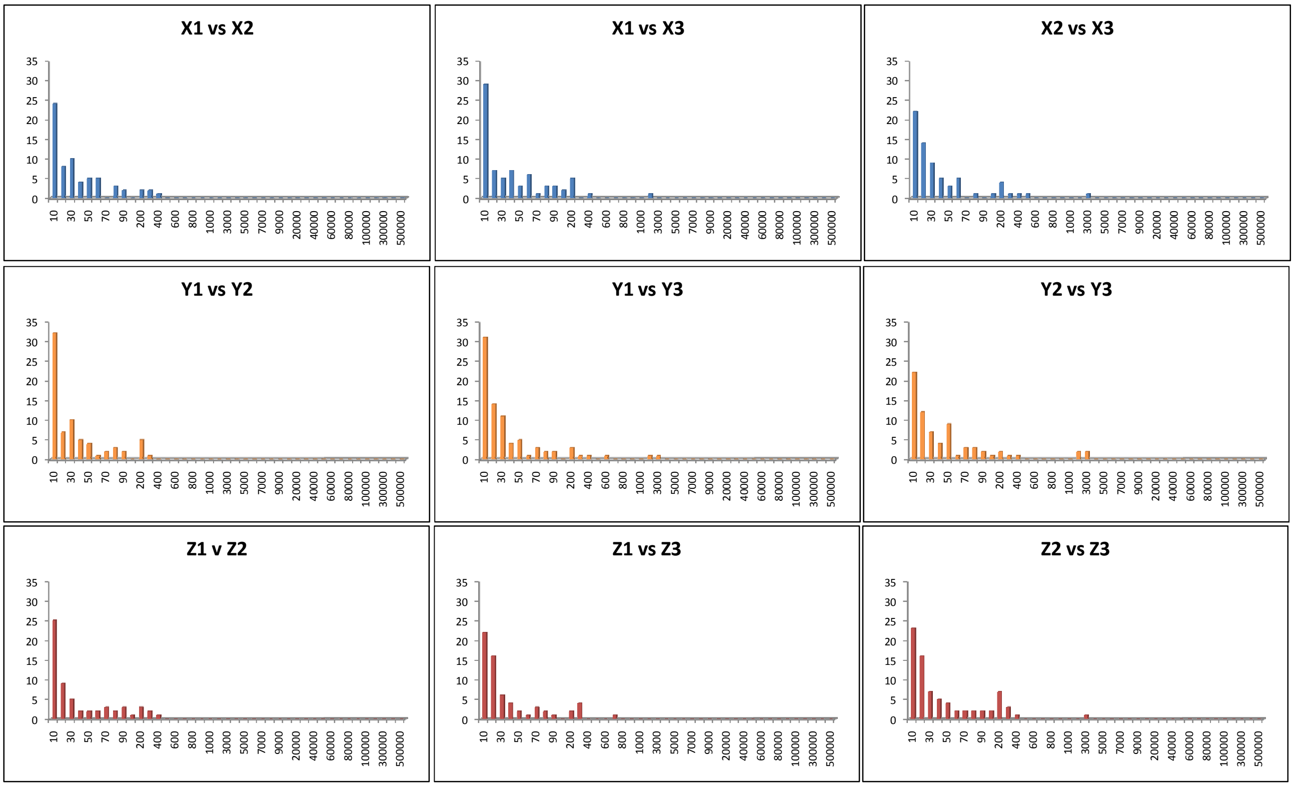

Figure S3. Representation of LOH events between genomes from the clonal isolates selected from the 3 strains (X, Y and Z). Panels a: Detection of large LOH event by chromosome. For each genome from clonal isolates, heterozygous SNPs density was mapped on the 8 chromosomes (1 Kb sliding windows). Homozygous regions are indicated in light or white colour. No appearance of large LOH event was observed between genome comparisons from clonal isolates by strain**.** The blue vertical line indicates the centromere of each chromosome. **Panels b: Density of LOH events by chromosome.** For each pair of genome comparison the starting location of all LOH events was mapped on the 8 chromosomes. For each pair-wise genome comparison LOH events were screened in symetric manner. (vs=versus, Chr= chromosome)

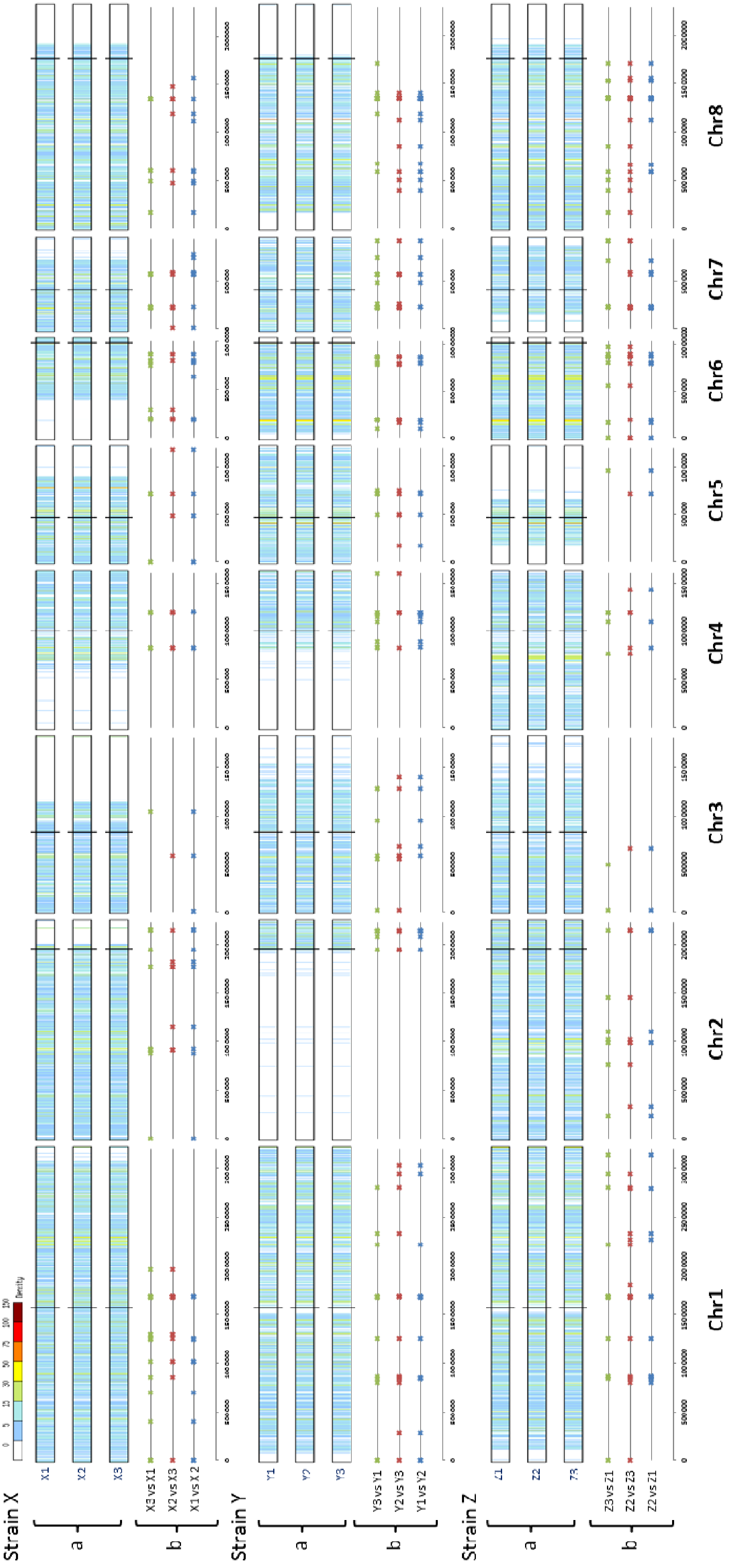
Strain X
a
X1
X2
X3
b
X3 vs X1
X2 vs X3
X1 vs X2
Strain Y
a
Y1
Y2
Y3
b
Y3 vs Y1
Y2 vs Y3
Y1 vs Y2
Strain Z
a
Z1
Z2
Z3
b
Z3 vs Z1
Z2 vs Z3
Z2 vs Z1
Density
Chr1
Chr2
Chr3
Chr4
Chr5
Chr6
Chr7
Chr8
